# Aging and health: Self-efficacy for Self-direction in Health Scale

**DOI:** 10.1590/S1518-8787.2016050006312

**Published:** 2016-06-28

**Authors:** Albertina L Oliveira, José T Silva, Margarida P Lima

**Affiliations:** Faculdade de Psicologia e Ciências da Educação. Universidade de Coimbra. Coimbra, Portugal

**Keywords:** Aged, Aging, Self-Efficacy, Self-Care, Personal Autonomy, Diagnostic Self-Evaluation, Diagnostic Techniques and Procedures

## Abstract

**OBJECTIVE:**

To validate the *Escala de Autoeficácia para a Autodireção na Saúde* (EAAS – Self-efficacy for Self-direction in Health Scale).

**METHODS:**

Non-experimental quantitative study of EAAS validation, by confirmatory factorial analyses, evaluating a sample of 508 older adults from the north and the center of Portugal with mean age of 71.67 (from 51 to 96 years), to whom the Self-efficacy for Self-direction in Health Scale, the Rosenberg Self-esteem Scale, the Positive and Negative Affect Schedule, the Satisfaction with Life Scale, and the Instrumental Activities of Daily Living Scale were applied. The EAAS was developed from the theoretical constructs of self-efficacy and from self-directed learning within the PALADIN European project framework, aiming to develop an instrument able to assess the extent to which older adults take good care of their health.

**RESULTS:**

The internal consistency was 0.87 (Cronbach’s alpha) and confirmatory factorial analyses enabled to find a model near the one theoretically proposed, indicating a structure consisting of four dimensions: physical exercise, healthy diet, engaging in health-related learning, and visits to health professionals. From the psychometric point of view, the model in four factors showed quite satisfactory fit indicators.

**CONCLUSIONS:**

The Self-efficacy for Self-direction in Health Scale, with 16 items, is adequate to evaluate to what extent older adults have confidence in their ability to take care of their own health, with high degree of autonomy.

## RESUMO

**OBJETIVO:**

Validar a Escala de Autoeficácia para a Autodireção no domínio da Saúde (EAAS).

**MÉTODOS:**

Estudo quantitativo não experimental de validação da EAAS, por meio de análises fatoriais confirmatórias, avaliando amostra de 508 seniores e idosos provenientes das regiões Norte e Centro de Portugal com média etária de 71.67 (51 a 96 anos), a quem foram aplicadas a Escala de Autoeficácia para a Autodireção na Saúde, a Escala de Autoestima de Rosenberg, a Escala de Afeto Positivo e Afeto Negativo, a Escala de Satisfação Com a Vida e a escala de Atividades Instrumentais da Vida Diária. A EAAS foi desenvolvida a partir dos construtos teóricos da autoeficácia e da aprendizagem autodirigida no âmbito do projeto europeu PALADIN, visando constituir um instrumento apto a avaliar até que ponto os seniores cuidam bem da sua saúde.

**RESULTADOS:**

A consistência interna encontrada foi de 0.87 (alfa de Cronbach) e as análises fatoriais confirmatórias permitiram encontrar um modelo próximo ao proposto teoricamente, indicando uma estrutura constituída por quatro dimensões: exercício físico, alimentação saudável, envolvimento em aprendizagens relativas à saúde e visitas a profissionais de saúde. Do ponto de vista psicométrico, o modelo em quatro fatores mostrou indicadores de ajustamento bastante satisfatórios.

**CONCLUSÕES:**

A Escala de Autoeficácia para a Autodireção na Saúde, com 16 itens, é adequada para avaliar em que medida os seniores têm confiança na sua capacidade para tomar conta da própria saúde, com elevado grau de autonomia.

## INTRODUCTION

The gerontological revolution in progress constitutes a great challenge to the change of several systems (social, political, economic, educational, and the health system, among others), especially in the area of health, to respond more effectively to the profound demographic changes that have been happening^27^. To preserve good health until advanced age is one of the best ways to ensure the quality of life of citizens, particularly in old age, when usually there are functional disabilities and a lesser degree of autonomy due to the degenerative processes that are occuring^1,13,26^. In this context, health care and the need to maintain a good functional state and independence are seen with great concern and personal and social ambition^24^. Since the decline may be slowed, to enhance the capacities of people and sensitize them to develop healthy lifestyles, preventing early deterioration and even reversing losses, is primordial^6,7,11^.

Self-direction in learning is a powerful factor against the premature decline and it strongly favors empowerment^18, 20, 21, a^. Self-directed older adults tend to have good control of their health with appropriate physical activity levels, maintaining a positive psychological state, managing specific health problems, and seeking to control their living environment. Overall, self-directed older adults assign great importance to learning, perceiving it as a mean to reduce the threats to their health, to increase their welfare, and to remain actively involved in the process of adaptation to the several changes that advanced age brings. Many older adults believe to be able to control their health and the aging process itself^3^, engaging themselves in strategies such as seeking for information about health (in television, newspapers, magazines, conversations, among others) and the compliance with the recommendations of health professionals regarding the practice of a healthy lifestyle. Taking good care of health does not exclude the active search for new learning and promotion of self-direction and balanced management of daily life on the dimensions that influence it the most^6,14,15^.

The need to enhance the health care of all citizens is also considered critical by the European Union within the 2020 strategy, which has defined as fundamental aim, for all member states, to increase in two years the healthspan, which starts establishing as effective priority the implementation of active ageing, defined as “the process of maximizing the potential of people to remain healthy, to participate in the life of their communities, and to improve their quality of life as they advance in age” (European Parliament Report, 2010, p. 25)^b^. In this context, the European Union supports projects in such a way that older adults and the structures designed for them may have innovative resources, such as the PALADIN project (Promoting Active Learning and Aging of Disadvantage Seniors), within which was developed the *Escala de Autoeficácia para a Autodireção na Saúde* (EAAS – Self-efficacy for Self-direction in Health Scale)^c^.

The development of instruments that allow evaluating to what extent older adults have confidence that they take good care of their health is of great utility. It was in this perspective that several instruments were conceived^d^, including the scale under study, starting with the constructs of self-efficacy^2,22^ and self-directed learning^17^. Regarding the first, according to Bandura^2^ (2006), self-efficacy is central to the understanding of human functioning, as it directly influences behavior and impacts other determinants, such as goals, aspirations, result expectations, perception of obstacles and opportunities that arise in the social environment. Regarding self-directed learning, studies consistently show that people with high levels of self-direction have personal initiative, are persistent, self-disciplined, and tend to be driven by objectives, as well as demonstrate high self-confidence, self-esteem, and satisfaction with life^8,16^.

In this context, to evaluate the day-to-day behaviors associated with good health and develop personal confidence in terms of control of these behaviors is essential for older adults to remain healthy throughout the aging process. Therefore, based on the specialty literature, as well as on professional and personal experience of the authors, we outlined the main dimensions underlying EAAS, which define the functioning domain relevant to health: physical exercise, diet, hygiene of life, contact with health professionals, and learning about health.

## METHODS

The process of construction of EAAS began by 10 focus group interviews with 23 older adults who attended day-care centers and new opportunities centers in Coimbra and its surroundings, having as main objective to identify their specific behaviors in the referred dimensions, the obstacles to be found, as well as previous attempts to change their condition. In addition, we tried to identify language, words, and expressions used, in such a way that the items of the scale could reflect a proper and accessible language to most older adults. The items generated in this process were submitted to the first test with the same people who had participated in the focal interviews and with older adults of a day-care center in Thessaloniki (Greece)^e^.

Having the instruments insufficiently validated within the PALADIN project, there was a need to study the EAAS more deeply, verifying construct validity and discriminant validity, by the correlation with Instrumental Activities of Daily Living (IADL)^12^, the Rosenberg Self-esteem scale (RSES)^25^, Positive Affect (PA) and Negative Affect (NA)^f^, the Satisfaction with Life Scale (SWLS)^23^, and age. Thus, 508 older adults were contacted in their homes, in local development associations, day-care centers, and new opportunities centers of the north and center of Portugal in 2013. All staff in data collection has received specific guidelines for the application of the instruments.

**Figure 1:**
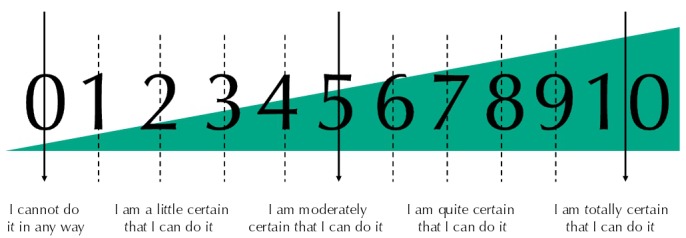
Response scale of Self-efficacy for Self-direction in Health Scale*.

The EAAS features on the first page an exercise of familiarization with the response scale, which varies between 0 (cannot do at all) and 10 (totally certain can do) and is colorful predominantly with navy blue color (to meet the aim of being attractive), containing the numbers in large size (Figure 1). Next, there are the instructions, the response scale, and its 22 items, 20 of which begin with “I am confident that I am able, by myself...”. The item 21, “Comparing your health with that of people of your age, how do you classify it?”, evaluates the subjective health and is answered on a five-point Likert scale, and the item 22, “Would you like to say something else about the confidence you have in yourself, regarding taking good care of your health?”, is an open item and finishes the scale. The first 20 items (of self-efficacy to self-direction) are distributed over five dimensions as follows: five items regarding physical exercise, six regarding diet, three regarding hygiene of life, three regarding consultations with health professionals, and three regarding learning about health.

* To access the complete range, see: The European Commission. PALADIN Project: Promoting Active learning and Ageing of Disadvantage Seniors. Available from: http://projectpaladin.eu

## RESULTS

The Portuguese sample was composed of 508 older adults, aged from 51 to 96 years (mean = 71.67; SD = 7.76). Concerning sex, 187 (36.8%) were men and 321 (63.2%), women. Mostly, they were married (52.8%) or widowed (29.5%). Regarding the level of education, 5.9% were illiterate, 9.6% could only read and write, 35.6% had between one and four years of study, 19.2% attended between five and nine years of school, 8.0% between 10 and 12 years, and 21.7% reported having post-secondary studies. Most (88.6%) were already retired when inquired, 4.5% indicated being employed, and 6.1% reported other condition.

The 508 older adults studied obtained in EAAS a mean of 142.56 (SD = 29.67), ranging between 57 and 200. In Table 1, we present descriptive statistics of the items of the scale and the respective values of the Cronbach’s alphas, which cause a global alpha of 0.87.

**Table 1:** Description of the items of Self-efficacy for Self-direction in Health Scale. (N = 508)

	Mean	SD	Asymmetry	SE	Skewness	SE	Item-total Correlation	Cronbach’s α if item deleted
Item 1	5.95	3.56	-0.47	0.108	-1.14	0.216	0.60	0.86
Item 2	8.97	2.82	-2.60	0.108	5.10	0.216	0.13	0.88
Item 3	7.02	2.73	-0.74	0.108	-0.40	0.216	0.58	0.86
Item 4	7.85	2.35	-1.05	0.108	0.46	0.216	0.41	0.87
Item 5	6.70	2.92	-0.77	0.108	-0.25	0.216	0.57	0.86
Item 6	7.68	2.35	-0.87	0.108	-0.02	0.216	0.31	0.87
Item 7	8.09	2.13	-1.16	0.108	0.89	0.216	0.39	0.87
Item 8	8.32	2.58	-1.75	0.108	2.40	0.216	0.25	0.87
Item 9	8.25	2.24	-1.29	0.108	0.80	0.216	0.53	0.86
Item 10	7.57	2.50	-0.98	0.108	0.12	0.216	0.38	0.87
Item 11	5.78	3.12	-0.36	0.108	-0.97	0.216	0.66	0.86
Item 12	8.49	1.93	-1.42	0.108	1.70	0.216	0.43	0.87
Item 13	7.33	2.67	-0.98	0.108	0.10	0.216	0.41	0.87
Item 14	6.39	3.25	-0.68	0.108	-0.71	0.216	0.69	0.85
Item 15	2.92	3.41	0.84	0.108	-0.68	0.216	0.32	0.87
Item 16	8.08	2.49	-1.36	0.108	1.01	0.216	0.47	0.86
Item 17	5.23	3.32	-0.18	0.108	-1.14	0.216	0.70	0.85
Item 18	8.00	2.45	-1.32	0.108	1.02	0.216	0.18	0.87
Item 19	5.86	3.25	-0.42	0.108	-0.98	0.216	0.70	0.85
Item 20	7.90	2.44	-1.26	0.108	0.89	0.216	0.54	0.86

Table 2 exposes different validity indicators obtained by the correlation coefficients between variables indicated in the Methods section.

**Table 2:** Indicators of construct validity and discriminant validity. (N = 508)

	Instruments					

	IADL	RSES	PA	NA	SWLS	Age
EAAS	0.397*	0.224*	0.384*	-0.326*	0.290*	-0.264*

EAAS: Self-efficacy for Self-direction in Health Scale; IADL: Instrumental Activities of Daily Living; RSES: Rosenberg Self-esteem Scale; PA: Positive Affect; NA: Negative Affect; SWLS: Satisfaction with Life Scale.

* p < 0.001.

Prior to the completion of the confirmatory factorial analysis (CFA), the existence of extreme values was evaluated by the Mahalanobis square distance (*D* ^2^) and the normality of variables was evaluated by the coefficients of univariate and multivariate asymmetry and skewness. No variable presented values of asymmetry (range: -0.29, 0.81) and skewness (range: -1.22, 6.67), indicators of severe violation to the normal distribution^10^. However, based on *D* ^2^ we found evidences of the presence of some extreme values, having removed 30 cases with statistically significant values. The evaluation of the presence of multicollinearity was analyzed using the tolerance and Variance Inflator Factor (VIF) statistics, having found no evidence of any problem (all values showed tolerance > 5 and VIF < 10). The model parameters of confirmatory factor analysis (regression coefficients, variances and covariances) were estimated by the maximum likelihood method, using AMOS software (v. 21, IBM SPSS Inc., Chicago, IL).

Regarding the results from the test of several structural models, the first two models were specified *a priori*, namely the theoretical design model originally idealized for the instrument (Model 1) and the model based on exploratory analysis of principal components (PC) previously performed^g^ (Model 2). These models correspond to a strictly confirmatory analytical modality to the extent they are proposed based on theoretical assumptions that precede the performance of any type of statistical analysis. Models subsequently tested, in turn, emerged from *a posteriori* changes suggested by the interpretation of the results, considering the indexes of modification available by the software used. In this sense, these models are respecifications of the original models, being inserted in a logic distinct from the previously mentioned, adopting a markedly exploratory nature.

Model 1 includes five latent variables: physical exercise (five items), healthy diet (seven items), hygiene of life (two items), learning about health (three items), and visits to health professionals (three items). On the other hand, Model 2 integrates four latent variables and features simplified empirical alternative of Model 1, and, to this end, it eliminated two items of healthy diet for presenting weak correlation with the other items of this factor; the hygiene factor of life was excluded because the insufficient number of items led to problems in the general identification of the model.

To evaluate the degree of fit of the hypothesized models, several indexes were used. Initially, we applied the goodness of fit Chi-square test^19^. However, since this test is highly affected by the complexity of the model and the size of the sample, we additionally used other indexes to better assess the quality of the fit. The Comparative Fit Index (CFI) confronts the hypothetical model with the null model, to ascertain if there was improvement. The CFI varies between 0 and 1, with values > 0.95 indicating good fit and values between 0.90-0.95, acceptable fit. The Normed Fit Index (NFI) and Tucker-Lewis Index (TLI) are also fit indicators and their values can be interpreted the same way as suggested for the CFI. Finally, the Root Mean Square Error of Approximation (RMSEA) was used to test the fit of the model, since it is less affected by sample size than the Chi-square. According to Hu and Bentler^9^ (1999), RMSEA values below 0.05 indicate a good fit, while values greater than 0.08 suggest poor fit. To compare the models between each other (for example, Model 1 *versus* Model 2), we used the Akaike Information Criterion (AIC), in the case of these models not being strictly nested and, otherwise, we used the test (of the difference) between the respective Chi-square values.

The test of the Model 1 has converged to an acceptable solution, resulting in Chi-square (N = 491, 160) = 636.9, p < 0.001. Although the value for absolute fit (evaluated by the statistics of Chi-square test) is statistically significant, suggesting the rejection of the null hypothesis (i.e., the model absolutely fits the data), this may be due, in part, to the large size of the sample used in the study. The analysis of some relative fit indexes, however, did not provide evidence of an adequate fit of the proposed model (for example, NFI = 0.86, TLI = 0.87, CFI = 0.89, RMSEA = 0.08, p < 0.001).

Model 2 also converged to an acceptable solution, having obtained a Chi-square (N = 491, 105) = 490.6, p < 0.001, suggesting a rejection of the null hypothesis and, consequently, an inadequate fit of the proposed model. Fit indexes confirm the misspecification of the model (NFI = 0.87, TLI = 0.88, CFI = 0.90, RMSEA = 0.09, p < 0.001). However, the relative comparison of the two models by AIC index (Model 1 AIC = 736.9 *versus* Model 2 AIC 560.6) shows that Model 1 is less parsimonious than Model 2.

Once the models tested are insufficiently fitted to data, we have sought to determine which are the main reasons for the observed misfit, based on the respecification of Model 2 for this analysis (the most parsimonious of the two). In this model, the items positively correlated with the respective latent variables (factors) and all the regression coefficients obtained were statistically significant (p < 0.001). The standardized regression coefficients ranged between 0.57-0.77, 0.77-0.87, and 0.29-0.79, respectively, for the healthy diet, physical activity, and health (this last factor resulting from the merger of the factors learning about health and visits to health professionals). Excluding the item “To search for information you need over the Internet”, belonging to the health factor, all presented coefficients greater than 0.50 (denoting good validity indexes). The intercorrelations between the three factors were positive and statistically significant: 0.30 (healthy diet *versus* physical exercise), 0.61 (physical exercise *versus* health), and 0.31 (healthy diet *versus* health).

The first respecification had as its starting point the observation that the regression coefficients “learning about health factors” and “visits to health professionals” were stronger when considered independent (Model 1) than when they appeared combined into a single factor (Model 2). Thus, we tested this new configuration (Model 2a), maintaining everything else the same as Model 2.

Model 2a has associated the Chi-square value (N = 491, 98) = 424.3, p < 0.001, in such a way that we conclude that, such as in previous tests, the nul hypothesis should be rejected. However, the other indicators of the fit degree showed some improvements: (NFI = 0.89, TLI = 0.89, CFI = 0.91, RMSEA = 0.08, p < 0.001). The AIC value for this model was lower than that recorded for the two models that had been analyzed (AIC = 500.2), and Model 2a preferred for being the more parsimonious one.

Psychometric properties of the indicators presented, in general terms, improvements. For instance, the regression coefficients of the items in the healthy diet, physical exercise, learning about health, and visits to health professionals factors ranged from 0.55-0.77, 0.78-0.87, 0.35-0.75, and 0.70-0.82, respectively. On the other hand, the interfactorial correlations, being all positive and statistically significant, varied between 0.25 (healthy diet and visits to health professionals) and 0.72 (learning about health and visits to health professionals). The remaining correlations were 0.30 (diet and exercise), 0.39 (healthy diet and learning), 0.56 (exercise and visits), and 0.62 (exercise and learning).

For the last respecification, we considered the values of the modification indexes, concerning the covariance between the indicators, which were cumulatively statistically significant (> 10.0) and plausible (for example, to admit the covariance of the errors of item 6, “To avoid fried food in my diet” and of item 7, “To have a diet based on cooked and grilled food” suffer covariance). In addition, the three items in the “learning about health” factor have shown to be empirically correlated with indicators of other factors (especially with those who understand the factors “healthy diet” and “physical exercise”). Since these intercorrelations seemed reasonable, they were considered in the respecification of the new model.

**Figure 2:**
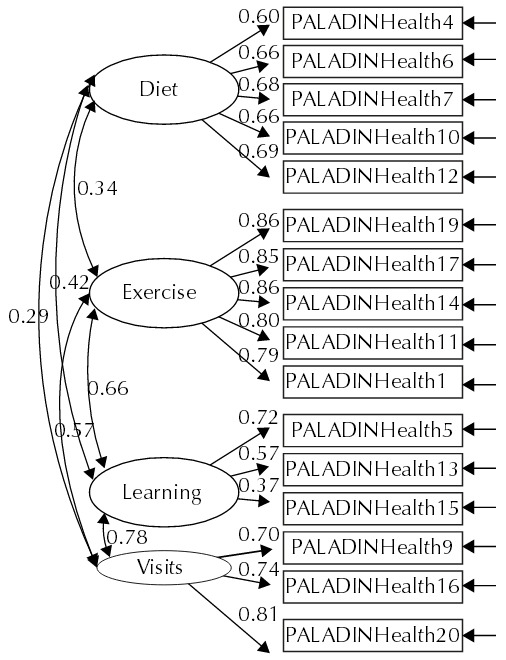
Final model of Self-efficacy for Self-direction in Health Scale.

This model (Model 2b) presented a Chi-square value (N = 491, 87) = 230.9, p < 0.001, which have not been perfectly fitted to data, but showed significant improvements in other fit factors to which we refer to: NFI = 0.94, TLI = 0.95, and CFI = 0.96. In particular, the RMSEA^4^ index indicated a very good fit of the model: RMSEA = 0.057 [0.05, 0.07], p = 0.09. Finally, the AIC value for this model is inferior to the one which was obtained for any of the previous models (AIC = 328.9), which makes it the most parsimonious among the analyzed. Figure 2 presents the diagram of trajectories with the standardized coefficients of the Model 2b.

Based on this final model, consisting of 16 items and four factors, we reestimated the internal consistency reliabilities and obtained a global Cronbach’s alpha of 0.87 (item-total correlations varying between 0.305 and 0.719). Regarding the reliabilities of the hypothesized dimensions, we found the following values: physical exercise (α = 0.92; five items; item-total correlations between 0.744 and 0.820); healthy diet (α = 0.80; five items; item-total correlations between 0.509 and 0.654); learning about health (α = 0.53; three items; item-total correlations between 0.239 and 0.454); and visits to health professionals (α = 0.80; three items; item-total correlations between 0.600 and 0.666).

## DISCUSSION

Here, we presented a scientifically valid scale, visually attractive and easy to use and understand.

Considering that there are different types of validity and that validation of an instrument is an unfinished process, it is necessary to gather several indicators of validity, as done in this study. Also considering the construct validity, which indicates the extent to which a measure has relationships with other variables according to theoretical predictions (Carmines and Woods, 2004)^5^, based on previous studies about self-directed learning and self-efficacy, EAAS was expected to present a correlation pattern consistent with the predictions. Effectively, the expected pattern of positive (with IADL, RSES, PA, and SWLS) and negative (NA and age) correlations were found and all results were significant, meaning that more older adults feel able to self-direct their health, have more autonomy in the activities of daily life, more perception of their self-esteem, of positive affect, and satisfaction with life. In contrast, the more negative affect experienced and older the person is, the lower the results in self-efficacy for self-direction in health. All correlations showed a median magnitude (Table 2).

Regarding reliability, the Cronbach’s coefficients obtained were, overall, quite suitable, both for global reliability and for the factors, with the exception of “learning about health” whose coefficient (α = 0.53) was weak. However, if we consider all the global alphas, in addition to being high, they are robust, since we know that the samples are associated with different cultures and population habits typical of several countries. The items of the scale seemed insensitive to these differences, allowing the evaluation of what is essential to take care of when we consider physical health.

Regarding factorial validity, though not confirming the original theoretical model, it was possible, with some changes, to obtain a model closed to this (contemplating four factors), that is, most dimensions theoretically expected based on literature review were effectively empirically found. The new configuration was obtained by eliminating two items of “healthy diet” (item 2: To abstain from consuming tobacco; item 8: To consume wine or other alcoholic beverages with moderation). We also had to suppress a factor initially proposed for the purposes of the confirmatory analyses, for it presented insufficient number of items (only two) and not for bad pyschometric behavior. It is suggested to increase the number of indicators for this factor in the future. “Learning about health” and “visits to health professionals” factors have remained separated, although they have shown substantial association between themselves (> 0.7). In this case, it is also suggested to increase the number of indicators (for example, to five).

In short, the final scale, consisting of 16 items, appears to have enough psychometric strength and adequacy for applied use in the field of health of older adults, more specifically when assessing to what extent these people have confidence in their ability to take care of their health, with a high degree of autonomy, involving themselves in very specific and concrete behaviors. However, the scale in this final version only evaluates physical exercise, diet, involvement in learning regarding health, and visits to health professionals.

Therefore, we conclude that the validity and reliability indicators evaluated pointed to good psychometric qualities of EAAS. Thus, the scale serves the purposes for which it was created, and may not only be used as an instrument for measuring of the level of self-efficacy for self-direction in health, but also for investigations in the field of health in which it is intended to assess to what extent older adults express confidence and autonomy in taking care of their health.

However, in further development of this measuring instrument, we suggest adding two dimensions (hygiene of life and abstention from harmful habits), in such a way that the functioning domain of the construct becomes more complete.

## Footnotes

a Valente JS. The role of self-directed learning in older adults’ health care [dissertation]. Athens (GA): University of Georgia; 2005.

b European Parliament. Report on the demographic challenge and solidarity between generations. Brussels: Committee on Employment and Social Affairs; 2010.

c The main objective of this project was to generate resources and instruments that could be made available for older adults to favor their capacity of taking control and having autonomy on central domains of life, such as health.

d The project’s webpage with the respective instruments is available from: *The European Commission. Project Paladin: Promoting Active learning and Ageing of Disadvantage Seniors*. Available from: http://projectpaladin.eu

e Oliveira AL, Vieira C, Lima MP, Nogueira S, Alcoforado L, Ferreira JA, et al. Developing instruments to improve learning and development of disadvantage seniors in Europe: The Paladin Project. In: Conference proceedings of the International Conference The Future of Education. Florence: Simonelli Editore; 2011. v.1, p.268-74.

f Watson D, Clark LA. The PANAS-X: manual for the positive and negative affect schedule - expanded form. Iowa: University of Iowa; 1994 [cited 2015 Nov 16]. Available from: http://www.psychology.uiowa.edu/faculty/Clark/

g Oliveira AL. A Escala de Auto-Eficácia para a Autodireção no domínio da saúde: estudos psicométricos. Comunicação apresentada no XII Congresso Internacional Galego-Português de Psicopedagogia; 11-13 set. 2013; Braga, Portugal.
